# Supplementary material for: Estimation of lifetime survival and predictors of mortality among TB with HIV co-infected children after test and treat strategies launched in Northwest, Ethiopia, 2021; a multicentre historical follow-up study
Source: PLoS One. 2021 Dec 21;16(12):e0258964. doi: 10.1371/journal.pone.0258964 (PMC8691625; doi:10.1371/journal.pone.0258964)
Supplement: Attachment — Submitted filename: Response to Reviewers.docx [file pone.0258964.s003.docx]

Manuscript ID: PONE-D-21-04696

Title: Estimation of Lifetime Survival and Predictors of Mortality Among TB with HIV Co-infected Children After Test and Treat Strategies Launched in South Gondar Public Hospitals, Northwest, Ethiopia, 2021; A Multicentre Historical Follow-up Study

**Dear professor Qigui Yu (PLOS ONE journal chief Editor)**

After going through the entire manuscript entitled "Estimation of Lifetime Survival and Predictors of Mortality Among TB with HIV Co-infected Children After Test and Treat Strategies Launched in South Gondar Public Hospitals, Northwest, Ethiopia, 2021; A Multicentre Historical Follow-up Study" you forwarded your constructive comments which we missed to touch. Therefore, we are glad enough to express our sincerest thanks for your constructive Editors and reviewers’ comment that could help improve novelty of our effort. Additionally, those comments are all valuable and very helpful for revising and improving our paper, as well as an important guiding to significance the article.

We hope you find our manuscript suitable for publication and look forward hearing from you. It is needless to state that we are happy to provide any more details in support of this manuscript. Hence, we are living in resource limited setting that unable access the international English language experts to write very clearly. The main corrections in the paper and the responds to the Editor’s and reviewer’s comments are as flowing. The revised portion are marked in the yellow highlighted in revised version manuscript.

***Yours sincerely,***

***Ermias Sisay (on behalf of all authors) Email: ermisis1888@gmail.com***

***Department of Pediatrics and Child Health Nursing, College of Health Sciences, Debre Tabor University, Debre Tabor, Ethiopia***

**Reviewer #1:**

**Comments #1:** In this study, the authors performed institution-based retrospective follow-up study conducted from March 1, 2014, to January 12, 2021 on survival and predictors of mortality among 227 TB and HIV co-infected children in Ethiopia. They found that this population of children had a high mortality rate of 17% and that cotrimoxazole preventive therapy (CPT) non-users, ART treatment failure, and CD4 count below threshold were significant predictors of mortality. The study was well done, but the writing needs substantial improvement.

Author’s response: We are grateful for your appreciation of the educative being of our study.

From repeated proof-reading of the manuscript, we found several grammatical errors, interlinings, police titles, punctuation errors, wordings and spelling errors. Therefore, finding our colleague who has Master of Arts in English, we have trying our best to thoroughly copyedit the manuscript for English language usage. These changes are found throughout the revised version manuscript.

**Comments #2**: There are no other major concerns. The following are some minor concerns and questions.

Author’s response: Great thanks! Having this comment: We revised your concern point by point that can be displayed in the revised version of the manuscript.

**Comments #3**: 1) Please provide explanation to the test and treat strategies and ART service

Author’s response: Test-and-treat is an intervention strategy in which the population at risk is screened for HIV infection and diagnosed HIV infected individuals receive early treatment, aiming to eliminate HIV as it reduces the rate of spreading the virus to other people. In Ethiopia test and treat strategies in children were started since in 2014 G.C. Hence, the purposes of this study were life time survival among TB & HIV co-infection what seem like during the test and treat strategies era

**Comments #4**: 2) Does duration on ART mean that the following time of the patients during the study.

Author’s response: Absolutely!

**Comments #5**: 3) How many patients in Stage III & IV were in Stage IV? Would Stage IV be a predictor of mortality?

Author’s response: In genuinely speaking,	161 patients were Stage III and 66 patients were	stage IV. However, we including stage I &II that is typing errors since all patient were TB and HIV co-infected children so that the children were stage III or IV. Sorry for including these confusing and incomplete statements once and again and amendment can be seen in the revised version of the manuscript specifically in the result section.

**Comments #6**: 4) In Table 3 CPT, the Yes and No groups were mislabeled.

Author’s response: We are missed to touch on this comment sir! We are revised based on the given comments.

**Comments #7**: 5) The Figure legends for Kaplan-Meier survival curves of CD4 counts are mixed-up. The legends for CPT user and non-user were the same.

Author’s response: Undoubtedly! Hence, it is modified based on the given comments. These changes are found throughout the revised version manuscript in figure submission and labeled at the end of the manuscript

**Comments #8**: 6) What were the major causes of death?

Author’s response: We strongly agree with the essence of incorporating this comment. According to this study, the major causes of death among TB&HIV co-infected children were the presence of Treatment failure, CD4 count below the threshold, WHO stage IV and cotrimoxazole preventive therapy non-users. Hence, screening early and managing this predictor variable can be reduce the risk of mortality significantly.

**Reviewer #2:**

**Comments #1:** The manuscript "Estimation of Lifetime Survival and Predictors of Mortality Among TB with HIV Co-infected children After Test and Treat Strategies Launched in South Gondar Public Hospitals, Northwest, Ethiopia, 2021; A Multicentre Historical Follow-up Study" by Chanie et al touched a very important heath issue among the HIV/TB co-infected people. However, the studies are not very organized so its enthusiasm is reduced.

Author’s response: Sure! From repeated proof-reading of the manuscript, we found several grammatical errors, interlinings, police titles, punctuation errors, wordings and spelling errors. Therefore, finding our colleague who has Master of Arts in English, we have tried our best to thoroughly copyedit the manuscript for English language usage. These changes are found throughout the revised version manuscript

Major concerns:

**Comments #2**. language: The whole paper is very hard to understand, current wiring is very sloppy.

Author’s response: Undoubtedly! We are modified the manuscript to be easily readable consulting English language experts. These improvements are found throughout the revised version manuscript.

**Comments #3:** Statistical analysis is lacking for the Tables 1 and 2. Although the statistical power (p value) was shown for the Table 3, it lacks a description of how the authors did the statistical analysis.

Author’s response: We are not quite sure on understanding on your comment sir. Tables 1 and 2 shows the proportion of Socio-demographic characteristics and Clinical and treatment-related characteristics. While, Table 3 shows the bivariable cox-proportional hazard models were employed for each predictor variable. Additionally, those variables having p-value < 0.25 in bivariate analysis were fitted into a multivariable cox-proportional hazards model. P-value < 0.05 was used to declare a significant association with the dependent variable. The p-values displayed in table 3 was shows that p-values with respect to each variable that only entered into multivariable only.

**Comments #4:** As the authors concluded "In this study, mortality among TB and HIV co-infected children was found to be very high. Treatment failure and CD4 count below the threshold were the independent predictors of mortality. Hence, it is better a special emphasis on monitoring and managing regularly of these contributing factors. Besides, strengthen the WHO recommendation of cotrimoxazole preventive therapy for all patients living with HIV is crucial." it is not novel at all, e.g., people have known the importance of CD4 count.

Author’s response: Yes indeed! We are revised based on the given comment. These changes are found in conclusion section of the revised version manuscript.

**Comments #5:** suggest the authors making Bar graphs for Table 1 and Table 2 to show more detailed data in clearer manner.

Author’s response: Unquestionably! We found it a logical comment; thus, it has been added the Bar graphs for only Table 2 to show more detailed data in clearer manner. Since according to plose one journal it is not recommend more than 0f figures. These revisions are displayed in result section of the revised version manuscript.

***Yours sincerely,***

***Ermias Sisay (on behalf of all authors) Email: ermisis1888@gmail.com***

***Department of Pediatrics and Child Health Nursing, College of Health Sciences, Debre Tabor University, Debre Tabor, Ethiopia***
