# Supplementary material for: Estimation of lifetime survival and predictors of mortality among TB with HIV co-infected children after test and treat strategies launched in Northwest, Ethiopia, 2021; a multicentre historical follow-up study
Source: PLoS One. 2021 Dec 21;16(12):e0258964. doi: 10.1371/journal.pone.0258964 (PMC8691625; doi:10.1371/journal.pone.0258964)
Supplement: Attachment — Submitted filename: PONE-D-21-04696R1.docx [file pone.0258964.s004.docx]

Comments to the Authors

Title: Estimation of Lifetime Survival and Predictors of Mortality Among TB with HIV Co-infected Children After Test and Treat Strategies Launched in South Gondar Public Hospitals, Northwest, Ethiopia, 2021; A Multicentre Historical Follow-up Study (*PONE-D-21-04696R1*)

The major issue had been poor English writing, which was improved in this revised version. However, there are still many grammar mistakes and the overall flow of the sentences need to be fixed. There are also some misstatements and typos, some of which are listed below.

The statement in the Abstract “However, there is no prior evidence in Ethiopia.” is not accurate as the authors themselves cited a reference to show the opposite (ref #18, Survival and predictors of mortality among children co-infected with tuberculosis and human immunodeficiency virus at University of Gondar Comprehensive Specialized Hospital, Northwest Ethiopia. A retrospective follow-up study. PLoS ONE [Internet]. 2018 May 22”). It appears that this current study and the above-mentioned 2018 study both recruited patients from the same Gondar Comprehensive Specialized Hospital. Were there any patients included in both studies? Some discussion on the similarities and differences between both studies would be helpful.

As the risk of mortality is associated with CPT therapy non-users, the concluding should emphasize CPT use along with ART therapy.

Some of the references need to be updated. For example, World Health Organization. Global tuberculosis report 2020 is available (ref #4); The data shown in the sentences “In 2015 worldwide, 1.6 million people…” should be replaced with more recent data.

Should the overall mortality rate be 3.7 per 100 person-years instead of 3.7 per person-years.

Should Debre Tabor compressive specialized hospital be Debre Tabor Comprehensive Specialized Hospital?

In “by taking sex as a predictor variable (on the male as the exposed group

denoted by q1 (0.38) and female group denoted by q0 (0.53)”, where the numbers come from?

The sentence “The patients provided informed written consent to have data/samples from their medical records used in research.” needs re-wording as they were too young to provide such consent.

In “half of 117 (51.54% and 122(53.74%) male and age between 6.10 years respectively.”, the order was wrong: 122 is for male and 117 for the age group.

In “118 (51.98%), 99 (43.61%), 103 (45.37%), and 218 (77.03%)…), what 218 is for?
