# Supplementary material for: Estimation of lifetime survival and predictors of mortality among TB with HIV co-infected children after test and treat strategies launched in Northwest, Ethiopia, 2021; a multicentre historical follow-up study
Source: PLoS One. 2021 Dec 21;16(12):e0258964. doi: 10.1371/journal.pone.0258964 (PMC8691625; doi:10.1371/journal.pone.0258964)
Supplement: Attachment — Submitted filename: Response to Reviewers.docx [file pone.0258964.s005.docx]

**Manuscript. Number.: PONE-D-21-04696R1**

**Title:** Estimation of Lifetime Survival and Predictors of Mortality Among TB with HIV Co-infected Children After Test and Treat Strategies Launched in Northwest, Ethiopia, 2021; A Multicentre Historical Follow-up Study

**Dear Dr Qigui Yu, M.D./Ph.D (PLOS ONE Chief Academic Editor) and Reviewers:**

After going through the entire manuscript, you forwarded your constructive comments which we missed touch. Therefore, we are glad enough to express our sincerest thanks for your constructive comments that could help improve the novelty of our effort. Those comments are all valuable and very helpful for revising and improving our paper, as well as the important guiding significance to our researches. We have studied comments carefully and have made a correction which we hope meet with approval. Revised portions are marked in the Yellow highlighted in the revised version manuscript. We hope you find our manuscript suitable for publication and look forward to hearing from you. It is needless to state that we are happy to provide any more details in support of this manuscript. The main corrections in the paper and the responses to the Editor's and reviewer's comments are as flows

***Yours sincerely,***

***Ermias Sisay Chanie (on behalf of all authors)***

***Department of Pediatrics and Child Health Nursing, College of Health Sciences, Debre Tabor University, Debre Tabor, Ethiopia***

**Point by point Author’s response to reviewer question and comment**

***Reviewer#1:***

**Comments #1**: The major issue had been poor English writing, which was improved in this revised version. However, there are still many grammar mistakes and the overall flow of the sentences need to be fixed. There are also some misstatements and typos, some of which are listed below.

Author’s response: Undoubtedly! It is modified based on the given comment.

After frequent proofreading of the manuscript had several grammatical wordings and spelling errors. Hence, based on the given comment revision was made. These changes are found throughout the manuscript. These changes are found throughout the revised version manuscript

**Comments #2**: The statement in the Abstract “However, there is no prior evidence in Ethiopia.” is not accurate as the authors themselves cited a reference to show the opposite (ref #18, Survival and predictors of mortality among children co-infected with tuberculosis and human immunodeficiency virus at University of Gondar Comprehensive Specialized Hospital, Northwest Ethiopia. A retrospective follow-up study. PLoS ONE [Internet]. 2018 May 22”). It appears that this current study and the above-mentioned 2018 study both recruited patients from the same Gondar Comprehensive Specialized Hospital. Were there any patients included in both studies? Some discussion on the similarities and differences between both studies would be helpful. As the risk of mortality is associated with CPT therapy non-users, the conclusion should emphasize CPT use along with ART therapy

Author’s response: We strongly agree with the essence of incorporating this comment!

Sure, we are stated that there is no prior evidence in Ethiopia regarding to estimation of lifetime survival and predictors of mortality among TB with HIV co-infected children after test and treat strategies launched in Northwest, Ethiopia, 2021. What makes this research differ from the previous one (i.e., ref #18 from 2005-2018) is: This study was conducted in after test and treat strategies launched (i.e., after 20014), recent evidence, and multicenter. However, we are concluded to revised as limited evidence than no evidence.

**Comments #3**: Some of the references need to be updated. For example, World Health Organization. Global tuberculosis report 2020 is available (ref #4); The data shown in the sentences “In 2015 worldwide, 1.6 million people…” should be replaced with more recent data.

Author’s response: Unquestionably! It is modified based on the given comment as Globally, there were 10 million people with TB in 2020, 1.2 million deaths from HIV-negative people and an additional 208,000 deaths from HIV-positive individuals.

**Comments #3**: Should the overall mortality rate be 3.7 per 100 person-years instead of 3.7 per person-years.

Author’s response: Yes indeed! It is amended based on the given comment

**Comments #4**: Should Debre Tabor compressive specialized hospital be Debre Tabor Comprehensive Specialized Hospital?

Author’s response: Sure! it has been modified based on the given comment

**Comments #5**: In “by taking sex as a predictor variable (on the male as the exposed group

denoted by q1 (0.38) and female group denoted by q0 (0.53)”, where the numbers come from?

Author's Response: Absolutely! we found this comment with the greatest relevance because it helps to convince readers who ask the same question after reading this paper.

The sample size can be determined by considering the previous study by taking its proportion/prevalence, incidence and associated/predictor factors. In this case, the sample size was determined based on the second objective (i.e., predictors. sex) from the previous study as we tried to cite after categorized as the probability of survival in exposed and non-exposed groups.

**Comments #6**: The sentence “The patients provided informed written consent to have data/samples from their medical records used in research.” needs re-wording as the patients were too young to provide such consent.

Author’s response: Accepted! In genuinely speaking, the informed written consent was obtained from ART focal person in each hospital on behalf of the study participants. Since it is secondary data. Hence, it is modified based on the given comment

**Comments #7**: In “half of 117 (51.54% and 122(53.74%) male and age between 6.10 years respectively.”, the order was wrong: 122 is for male and 117 for the age group.

Author’s response: Appreciated! Hence, it is revised based on the given comment!

**Comments #8**: In “118 (51.98%), 99 (43.61%), 103 (45.37%), and 218 (77.03%) …), what 218 is for?

Author’s response: Undoubtedly! it is removed since it is a typing error.

***Reviewer #3:***

**Comments #1**: The authors studied lifetime survival and predictors of mortality in children with TB and HIV coinfection. The overall mortality rate was 3.7, which as associated with treatment failure, especially CD4 count below the threshold. The manuscript has improved adequately, but there are minor concerns that should be made clear for the conclusion in relation to treatment failure.

Author’s response: Great thanks for having this comment and concern in the following statements!

**Comments # 2.** Antiretroviral drug (ARV) combination in the patient cohorts and groups, especially those with treatment failure, since 18.94% co-infected children showed treatment failure;

Author’s response: We were categorized the study participants in a group at the very beginning of the study in the different predictor variables. In this case, the baseline of the study participants (i.e., co-infected children) regarding to treatment failure was grouped. and then we follow them up to the interesting outcome (i.e., death) development or censored.

**Comments #3.** Did the ART cessation happens in the co-infected patients, which likely contribute to treatment failure, such as CD4 decline to the levels of threshold and emergence of opportunistic infection.

Author’s response: Sure! You know and we know that if TB and HIV co-infected children have treatment failure, the risk of CD4 decline and the emergence of opportunistic infection increased.

In the meantime, CD4 decline and emergence of opportunistic infection can increase the risk of treatment failure. So that, we were categorized the study participant via different predictor variables of the dependent variable at the entrance of the study, which implies in controlling the confounding effect of one predictor variable on another predictor (the effect of treatment failure over CD4 count and OI to towards mortality).

**Comments #4.** These are important to make statistical analysis and conclusion; The ARV combination and treatment duration in cohort with treatment failure should be considered in the group classification in the statistical analysis, at least discussed.

Author’s response: Appreciated! In this study, the effect of ARV combination (i.e. EFV-based

NVP, and PI) and treatment duration (<60 months vs ≥60 months) for the outcome variable was assessed/analyzed. However, it is not discussed and analysed with treatment failure. Hence, it is discussed the revised version manuscript in the discussion section that can be can be seen from in yellow highlighted manuscript.

***Thank you with best regards.***

***Yours sincerely,***

***Ermias Sisay Chanie (on behalf of all authors)***

***Department of Pediatrics and Child Health Nursing, College of Health Sciences, Debre Tabor University, Debre Tabor, Ethiopia***
