# Supplementary material for: Estimation of lifetime survival and predictors of mortality among TB with HIV co-infected children after test and treat strategies launched in Northwest, Ethiopia, 2021; a multicentre historical follow-up study
Source: PLoS One. 2021 Dec 21;16(12):e0258964. doi: 10.1371/journal.pone.0258964 (PMC8691625; doi:10.1371/journal.pone.0258964)
Supplement: Attachment — Submitted filename: Response to Reviewers.docx [file pone.0258964.s006.docx]

**Manuscript. Number.: PONE-D-21-04696R1**

**Title:** Estimation of Lifetime Survival and Predictors of Mortality Among TB with HIV Co-infected Children After Test and Treat Strategies Launched in Northwest, Ethiopia, 2021; A Multicentre Historical Follow-up Study

**Dear Dr Qigui Yu, M.D./Ph. D (PLOS ONE Chief Academic Editor)**

We appreciate you and the reviewers for your precious time in reviewing our paper and providing valuable comments. It was your valuable and insightful comments that led to possible improvements in the current version. The authors have carefully considered the comments and tried our best to address every one of them. We hope the manuscript after careful revisions meet your high standards. The authors welcome further constructive comments if any.

Below we provide the point-by-point responses. All modifications in the manuscript have been highlighted in yellow.

***Yours sincerely,***

***Ermias Sisay Chanie (on behalf of all authors)***

***Department of Pediatrics and Child Health Nursing, College of Health Sciences, Debre Tabor University, Debre Tabor, Ethiopia***

**Point by point Author’s response to Editors and reviewers**

**Editors**

**[General Comment]** Please review your reference list to ensure that it is complete and correct. If you have cited papers that have been retracted, please include the rationale for doing so in the manuscript text, or remove these references and replace them with relevant current references. Any changes to the reference list should be mentioned in the rebuttal letter that accompanies your revised manuscript. If you need to cite a retracted article, indicate the article’s retracted status in the References list and also include a citation and full reference for the retraction notice.

**Response:** Thank you very much for the reminder. We have made revisions accordingly.

**[General Comment]** Suggest to seek professional English editing services to improve the flow and writing of your manuscript.

**Response:** In genuine speaking, we can’t obtain native English language experts to revised the manuscript intensively since we are living in highly resource limited setting. However, we have carefully considered the comments and tried our best to address every one of them to revised and to made the manuscript beauty.

**[General Comment]** If reviewer comments were submitted as an attachment file, they will be attached to this email and accessible via the submission site. Please log into your account, locate the manuscript record, and check for the action link "View Attachments". If this link does not appear, there are no attachment files.]

**Response:** We are needless to state that we are happy to provide any more details in support of this manuscript as required.

We are observed the attachment file in action link, and there is one attachment file. However, all comments and suggestion in the attached file was addressed in the first round. Now, we are attached once ana again as follows.

***Reviewer#1:***

**Comments #1**: The major issue had been poor English writing, which was improved in this revised version. However, there are still many grammar mistakes and the overall flow of the sentences need to be fixed. There are also some misstatements and typos, some of which are listed below.

Author’s response: Undoubtedly! It is modified based on the given comment.

After frequent proofreading of the manuscript had several grammatical wordings and spelling errors. Hence, based on the given comment revision was made. These changes are found throughout the manuscript. These changes are found throughout the revised version manuscript

**Comments #2**: The statement in the Abstract “However, there is no prior evidence in Ethiopia.” is not accurate as the authors themselves cited a reference to show the opposite (ref #18, Survival and predictors of mortality among children co-infected with tuberculosis and human immunodeficiency virus at University of Gondar Comprehensive Specialized Hospital, Northwest Ethiopia. A retrospective follow-up study. PLoS ONE [Internet]. 2018 May 22”). It appears that this current study and the above-mentioned 2018 study both recruited patients from the same Gondar Comprehensive Specialized Hospital. Were there any patients included in both studies? Some discussion on the similarities and differences between both studies would be helpful. As the risk of mortality is associated with CPT therapy non-users, the conclusion should emphasize CPT use along with ART therapy

Author’s response: We strongly agree with the essence of incorporating this comment!

Sure, we are stated that there is no prior evidence in Ethiopia regarding to estimation of lifetime survival and predictors of mortality among TB with HIV co-infected children after test and treat strategies launched in Northwest, Ethiopia, 2021. What makes this research differ from the previous one (i.e., ref #18 from 2005-2018) is: This study was conducted in after test and treat strategies launched (i.e., after 20014), recent evidence, and multicenter. However, we are concluded to revised as limited evidence than no evidence.

**Comments #3**: Some of the references need to be updated. For example, World Health Organization. Global tuberculosis report 2020 is available (ref #4); The data shown in the sentences “In 2015 worldwide, 1.6 million people…” should be replaced with more recent data.

Author’s response: Unquestionably! It is modified based on the given comment as Globally, there were 10 million people with TB in 2020, 1.2 million deaths from HIV-negative people and an additional 208,000 deaths from HIV-positive individuals.

**Comments #3**: Should the overall mortality rate be 3.7 per 100 person-years instead of 3.7 per person-years.

Author’s response: Yes indeed! It is amended based on the given comment

**Comments #4**: Should Debre Tabor compressive specialized hospital be Debre Tabor Comprehensive Specialized Hospital?

Author’s response: Sure! it has been modified based on the given comment

**Comments #5**: In “by taking sex as a predictor variable (on the male as the exposed group

denoted by q1 (0.38) and female group denoted by q0 (0.53)”, where the numbers come from?

Author's Response: Absolutely! we found this comment with the greatest relevance because it helps to convince readers who ask the same question after reading this paper.

The sample size can be determined by considering the previous study by taking its proportion/prevalence, incidence and associated/predictor factors. In this case, the sample size was determined based on the second objective (i.e., predictors. sex) from the previous study as we tried to cite after categorized as the probability of survival in exposed and non-exposed groups.

**Comments #6**: The sentence “The patients provided informed written consent to have data/samples from their medical records used in research.” needs re-wording as the patients were too young to provide such consent.

Author’s response: Accepted! In genuinely speaking, the informed written consent was obtained from ART focal person in each hospital on behalf of the study participants. Since it is secondary data. Hence, it is modified based on the given comment

**Comments #7**: In “half of 117 (51.54% and 122(53.74%) male and age between 6.10 years respectively.”, the order was wrong: 122 is for male and 117 for the age group.

Author’s response: Appreciated! Hence, it is revised based on the given comment!

**Comments #8**: In “118 (51.98%), 99 (43.61%), 103 (45.37%), and 218 (77.03%) …), what 218 is for?

Author’s response: Undoubtedly! it is removed since it is a typing error.
